# Functional outcomes of protocol-based rehabilitation for patients with coronavirus disease 2019 in an acute care setting

**DOI:** 10.20407/fmj.2023-015

**Published:** 2023-11-29

**Authors:** Yoshitaka Wada, Seiko Shibata, Ayato Shinohara, Koji Mizutani, Masahiko Mukaino, Yohei Otaka

**Affiliations:** 1 Department of Rehabilitation Medicine I, School of Medicine, Fujita Health University, Toyoake, Aichi, Japan; 2 Department of Rehabilitation, Fujita Health University Hospital, Toyoake, Aichi, Japan

**Keywords:** Coronavirus disease 2019, SARS-CoV-2, Rehabilitation, Exercise therapy, Intensive care unit

## Abstract

**Objectives:**

To examine the functional outcomes of patients with coronavirus disease 2019 (COVID-19) who underwent a new protocol-based rehabilitation program.

**Methods:**

In this retrospective cohort study, we enrolled patients who were hospitalised in a university hospital in Japan because of COVID-19 from 1^st^ September, 2020–5^th^ July, 2021. The primary outcome was the Functional Independence Measure (FIM) subtotal score for motor items at discharge. The secondary outcomes included the FIM cognitive subtotal score, length of hospital stay, rehabilitation period, total rehabilitation time, final rehabilitation protocol level, and discharge destination.

**Results:**

Of the 78 enrolled patients (49 men; mean age [standard deviation], 70.3 [13.9] years), 24 died (30.8%) during hospitalisation. Disease severity was classified as mild, moderate I, moderate II, and severe in 1, 6, 41, and 30 patients, respectively. The FIM motor subtotal score differed significantly among groups for all participants (p=0.027). Post hoc analysis revealed that the FIM motor subtotal score in the severe group was significantly lower than that in the moderate II group (p=0.030).

**Conclusions:**

Disease severity significantly affected patients’ functional outcome for COVID-19 at discharge. Our protocol-based program provides a benchmark for COVID-19 rehabilitation in an acute care setting.

## Introduction

Coronavirus disease 2019 (COVID-19), caused by the severe acute respiratory syndrome coronavirus 2 (SARS-CoV-2), emerged in December 2019 and continues to have a significant impact worldwide.^1^ A study from China in the early stage of the epidemic reported that 14% of COVID-19 patients experienced a severe form of the disease, and 5% were critical.^2^ Patients with COVID-19 are at risk of muscle weakness, motor impairment, joint stiffness, and pain caused by prolonged bed rest.^3^ Thus, there is a strong need for rehabilitation. However, the most effective rehabilitation methods for patients with COVID-19 remain uncertain.^4^

Early rehabilitation is recommended, including in critically severe cases,^5–7^ although a defined rehabilitation protocol has not yet been established. A rehabilitation protocol for patients with COVID-19 has been reported in an intensive care unit (ICU) study,^8^ which proposed two intervention programs: one for patients with a fraction of inspired oxygen (FiO2) ≥40% and <60%, and one for those with FiO2 ≥21% and <40%. At our centre, we have developed our own COVID-19 rehabilitation protocol, which includes more detailed steps depending on the patient’s status.

Few studies^9,10^ have reported activities of daily living (ADLs) at discharge for patients with COVID-19 who underwent rehabilitation during hospitalisation. Therefore, the effects of rehabilitation with a detailed protocol have not yet been confirmed. To establish more suitable COVID-19 rehabilitation programs, the ADL outcomes that can be achieved at discharge by rehabilitation using a unified protocol should be elucidated. Therefore, examining the functional outcomes of patients with COVID-19 undergoing rehabilitation is necessary.

Herein, we aimed to examine the functional outcomes of patients with COVID-19 who underwent protocol-based rehabilitation at an acute care hospital in Japan.

## Methods

This was a retrospective cohort study conducted at Fujita Health University Hospital, Aichi, Japan, a university hospital with 1,376 beds. This study was approved by the Ethics Committee of Fujita Health University (HM21-258). The requirement for informed consent was waived because of the retrospective study design, and all individuals who did not opt-out were therefore included.

### Participants

Patients who were hospitalised with a diagnosis of COVID-19 underwent rehabilitation based on a protocol developed in our hospital in the ICU and acute care wards and discharged between 1^st^ September, 2020 and 5^th^ July, 2021, were enrolled. The observation periods were during Japan’s third (from November 2020 to February 2021) and fourth (from March 2021) COVID-19 pandemic waves,^11^ during which the alpha and delta SARS-CoV-2 variants were dominant.^12^ Patients were classified as severe (requiring a ventilator), moderate II (with respiratory failure), moderate I (without respiratory failure), or mild, according to the “Guidelines for the treatment of novel coronavirus infections” in Japan (Supplementary Table 1).^13^ Assessment of the severity of the patient’s illness was conducted at the time of admission.

### Rehabilitation protocol

We developed an early rehabilitation protocol on the basis of previous guidelines^14,15^ and reviews^16–18^ of COVID-19 rehabilitation published before January 2021. During the creation of the protocol, COVID-19 was a new disease, and we did not understand how it would progress. The aim of this protocol was to 1) minimise disuse associated with hospitalisation and treatment, 2) optimise the risk management during acute rehabilitation, 3) provide consistent protocols from the acute to subacute phase, and 4) understand how the disease progresses. The rehabilitation protocol is shown in Table 1.

This protocol was divided into six levels, which were applied on the basis of each patient’s status. Level 1 indicated patients undergoing sedation; these patients were maintained on ventilators and received extracorporeal membrane oxygenation (ECMO) and blood purification therapy. At this level, prone positioning, range-of-motion training, and neuromuscular electrical stimulation were performed to prevent disuse of the extremities. Level 2 defines patients as being able to maintain a sitting position. This includes raising the backrest and, in some instances, sitting on the edge of the bed. Ventilators, ECMO, and blood purification therapy were continued, and some patients underwent tracheostomy. During this level, pulmonary rehabilitation focused on chest range-of-motion training, and muscle strength training was performed to the limit where oxygen demand did not increase, and sitting on the bed was gradually advanced. Level 3 indicated patients requiring tracheostomy or oxygenation who were able to remain standing. At this level, training predominantly focused on pulmonary rehabilitation and muscle strength training. If the patient did not present with respiratory failure, backless sitting and balance training in the standing position were also performed. Level 4 indicated the time required to start walking slowly. Patients continued to stretch their respiratory muscles and strengthened their limb muscles to increase their walking distance without respiratory failure. Level 5 indicated the time to start aerobic exercise, and level 6 indicated the time to start moderate-intensity exercise. Each level had defined upper limits for respiratory rate and heart rate and acceptable values for percutaneous oxygen saturation and rate of perceived exertion, which was also the criteria for level transition. The indication and discontinuation of rehabilitation were determined by a physiatrist in consultation with the physician in charge. In the ICU, rehabilitation was initiated early from the time of sedation and intubation under the direction of the intensive care specialist.

Physiatrists prescribed physical therapy, occupational therapy, and speech-language-hearing therapy on the basis of the protocol. The transition to the next level was determined by the physiatrists and therapists in charge, in accordance with the criteria for level transition.

### Outcomes

The primary outcome was the Functional Independence Measure (FIM)^19,20^ motor subtotal score at discharge. The secondary outcomes included the FIM cognitive subtotal score, length of hospital stay, rehabilitation period, total rehabilitation time during hospitalisation, final level of the rehabilitation protocol, and discharge destination. These outcomes and baseline characteristics, including age, sex, body mass index, number of patients who were admitted to the ICU, and comorbidity, were collected retrospectively from medical records. The observation period was from admission to our hospital until discharge, long-term care facility, or transfer to another hospital.

The FIM is a scale for activities of daily living comprising 13 motor items and five cognitive items.^19,20^ The motor subtotal of the FIM score ranges from 13 to 91, while the cognitive subtotal score ranges from 5 to 35. A higher score indicates better activities of daily living. The validity and reliability of this scale have been previously confirmed.^21^

### Analyses

Baseline characteristics were compared between each severity level of COVID-19 using the Kruskal–Wallis test or chi-square test, depending on the variable type. The FIM motor and cognitive subtotal scores at discharge, length of hospital stay, the number of days of rehabilitation, total rehabilitation time, and the final level of the rehabilitation protocol achieved were compared among COVID-19 severity groups using the Kruskal–Wallis test. To minimise selection bias during enrollment,^22^ patients who died during the study were included and assigned scores of 13 for the FIM motor subtotal score and 5 for the FIM cognitive subtotal score at discharge. Differences in discharge destination were examined using the chi-square test on the basis of COVID-19 severity. When statistically significant differences in a certain variable were found using the Kruskal–Wallis test, multiple comparisons were performed using the Wilcoxon rank sum test with Bonferroni correction. Because only one case was categorised as mild, the comparison based on severity was performed among only the remaining three groups (moderate I, moderate II, and severe). For the subgroup analysis, we analysed the above-mentioned items for survivors in the same manner as that for total participants but only by excluding the deceased patients. Furthermore, to obtain a benchmark regarding the outcomes corresponding to the achieved final level of the protocol, the outcomes were analysed according to each final level of the protocol achieved in survivors. Regarding the comparison between the levels, four groups (levels 2, 3, 4, and 5) were compared, as levels 1 and 6 had only one case each. Any *p*-values less than 0.05 were considered to be statistically significant. IBM SPSS (version 26.0. Armonk, NY, IBM Corp) was used for statistical analyses.

## Results

### Baseline characteristics

Among 300 consecutive patients with COVID-19 admitted to the hospital during the study period, 137 were admitted to the ICU and acute care units. Among them, 78 patients (mean [standard deviation, SD] age: 70.3 [13.9] years; 49 men) underwent protocol-based rehabilitation and were enrolled in the analysis.

The baseline characteristics of the study participants are shown in Table 2. The 78 patients were stratified on the basis of COVID-19 severity into mild, moderate I, moderate II, and severe groups, with 1, 6, 41, and 30 patients in each group, respectively. One patient categorised as mild had chronic kidney disease and Parkinson’s disease, and, therefore, required hospitalisation because of concerns regarding progression to severe COVID-19. The number of patients admitted to the ICU was 63 (moderate II 33/severe 30). All COVID-19 patients requiring ventilation in the hospital were admitted to the ICU. The diagnosis of pneumonia was performed in all cases, except for one mild case. During hospitalisation, four cases suffered from cerebral infarction, and one suffered from lower extremity arterial thromboembolism. Of the 78 patients, 24 (30.8%) died, all of whom were categorised as moderate II or severe.

Age and body mass index were significantly different between groups. Patients’ age was significantly lower in the moderate II (72.2 [13.5]) and severe groups (63.4 [12.0]) compared with the moderate I group (85.7 [5.8]) (*p*=0.009 and 0.002, respectively). Body mass index was significantly higher (28.7 [6.7]) in the severe group compared with the moderate II (23.0 [6.3], *p*=0.002) and moderate I groups (18.8 [2.7], *p*=0.001) and was also higher in the moderate II group compared with the moderate I group (*p*=0.024). Risk factors for severe COVID-19, namely, hypertension, diabetes mellitus, and dyslipidaemia, were present in 39 (50.0%), 24 (30.8%), and 21 (26.9%) patients, respectively.

### Outcomes in all participants according to severity

The FIM motor subtotal score differed significantly among the groups (*p*=0.027). Post hoc analysis revealed that the FIM motor subtotal score in the severe group was significantly lower than that in the moderate II group (31.6 [30.4] vs. 54.2 [30.1], *p*=0.030) (Table 3).

**Figure 1:**
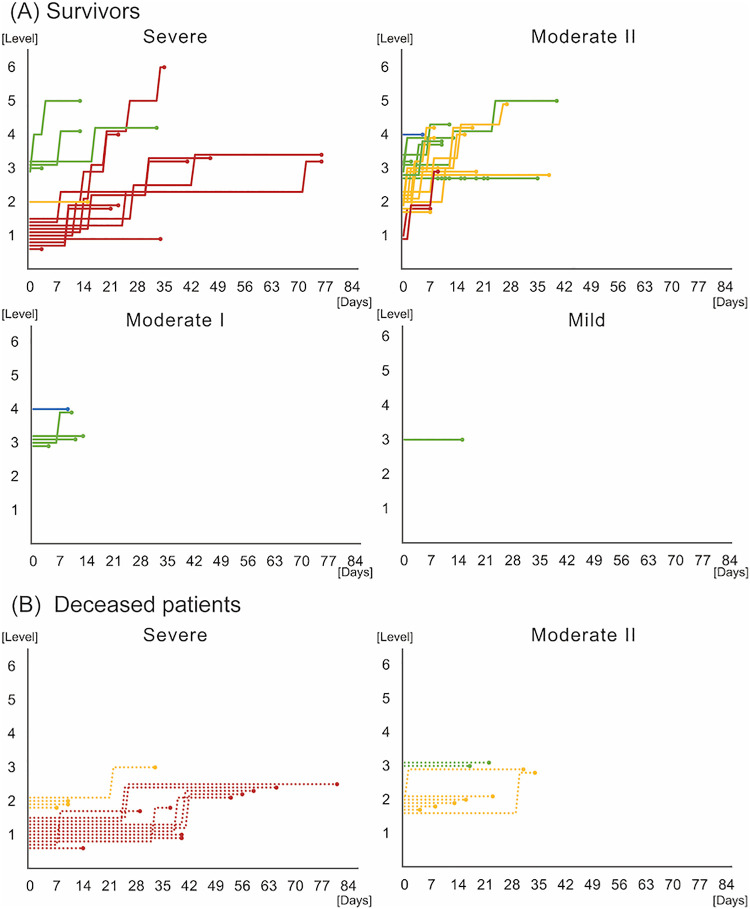
Rehabilitation protocol level progression according to disease severity The horizontal axis denotes the time required for rehabilitation in days. The vertical axis denotes the level of the rehabilitation protocol. The right-most circles indicate the protocol levels at discharge or final observation. Solid lines indicate survivors (A). Dotted lines indicate deceased patients (B). The red line indicates level 1, the yellow line indicates level 2, the green line indicates level 3, and the blue line indicates level 4 at the beginning of the rehabilitation.

The FIM cognitive subtotal score (*p*=0.026), length of hospital stay (*p*<0.001), days of rehabilitation period (*p*<0.001), total rehabilitation time (*p*<0.001), final level of rehabilitation protocol (*p*=0.002), and discharge destination (*p*=0.012) were significantly different among all of the groups. Post hoc analysis revealed that the FIM cognitive subtotal score in the severe group was significantly lower than that in the moderate II group (16.7 [12.9] vs. 25.3 [13.2], *p*=0.026). The length of hospital stay in the severe group (45.7 [17.7]) was significantly longer than that in the moderate II (25.8 [14.0], *p*<0.001) and moderate I groups (23.0 [8.3], *p*=0.004). The number of rehabilitation days in the severe group was significantly higher than that in the moderate II group (25.7 [18.5] vs. 11.1 [7.9], *p*<0.001). Total rehabilitation time in the severe group (1468 [1232.1]) was significantly longer than that in the moderate II (495 [425.3], *p*<0.001) and moderate I groups (286.7 [150.8], *p*=0.016). Patients in the moderate II group were likelier to be discharged home than those in the severe group (*p*=0.029). The individual-level progression of the rehabilitation protocol is shown in Figure 1. The severe group started from levels 1–3, the moderate II group from levels 2 and 4, the moderate I group from levels 3 or 4, and the mild group from level 3.

### Survivor outcomes according to severity

Supplementary Table 2 shows the baseline characteristics of the survivors. The FIM cognitive subtotal score at discharge (*p*=0.002), days of hospital stay (*p*=0.004), days of rehabilitation period (*p*=0.004), total rehabilitation time during hospital stay (*p*=0.002), and discharge destination (*p*=0.047) differed significantly among the groups (Table 4). The FIM motor subtotal scores at discharge (*p*=0.154) and the final level of the rehabilitation protocol (*p*=0.263) were not significantly different among the groups. Post hoc analyses revealed that the FIM cognitive subtotal score was significantly higher in the moderate II group than in the moderate I group (31.9 [7.4] vs. 20.0 [7.3], *p*=0.001). Furthermore, the length of hospital stay was significantly longer in the severe group (40.8 [17.5]) than in the moderate II (25.8 [14.1], *p*=0.007) and moderate I groups (23.0 [8.3], *p*=0.027). The total rehabilitation time was significantly longer in the severe group (1632.5 [1342.7]) than that in the moderate II (535.4 [459.4], *p*=0.006) and moderate I groups (286.7 [150.8], *p*=0.016). The discharge destination showed no significant difference in the post hoc analyses.

At the time of discharge, 12 patients (21.4%) required oxygen inhalation, 10 (severe 3/moderate II 6/moderate I group 1) required a nasal cannula, and three (severe group 3) required a ventilator. Among the survivors, there were no instances of reintubation or transfer from the general ward to the ICU following the start of the rehabilitation protocol.

### Survivor outcomes according to the final level achieved at discharge

A comparison of the FIM scores at each final level on the basis of the rehabilitation protocol is shown in Table 5. The FIM motor (*p*=0.004) and cognitive (*p*=0.014) subtotal scores, length of hospital stay (*p*=0.020), and total rehabilitation time (*p*=0.041) at discharge were significantly different among the groups. However, the rehabilitation period and discharge destination were not significantly different among the groups.

Post hoc analyses revealed that the FIM motor and FIM cognitive subtotal scores in the level 3 group were significantly lower than those in the level 4 group (51.3 [34.9] vs. 75.6 [15.1], *p*=0.037; 26.2 [9.9] vs. 33.7 [3.5], *p*=0.031). The total rehabilitation time showed no significant difference in the post hoc analyses.

## Discussion

In the current study, we investigated the FIM score at discharge, rehabilitation time, and discharge destination according to COVID-19 severity and the achieved level of our rehabilitation protocol. The findings demonstrated that the FIM motor subtotal score at discharge showed a significant difference among disease severity levels. Severity was a significant factor in deterioration in the functional outcome at discharge among patients hospitalised for COVID-19. However, the FIM motor subtotal score in survivors showed no significant difference among severity levels, indicating that patients with COVID-19 may achieve good functional outcomes, even in severe but survivable cases.

Overall, our findings indicate that the FIM motor subtotal score at discharge showed a significant difference among severities in all participants. This may have occurred because increased COVID-19 severity is likely to lead to poor prognosis.^23,24^ However, the Japanese severity classification for SARS-CoV-2 infection considers respiratory symptoms (particularly dyspnoea) and oxygenation, which differs from the classifications of the World Health Organization^25^ and the National Institutes of Health.^26^ Although the clinical symptoms of COVID-19 are not limited to respiratory symptoms, the results of the current study suggest that the Japanese severity classification may have practical utility in predicting prognosis. In contrast, the FIM motor subtotal score at discharge in survivors did not show significant differences among severities. Although selection bias for admission and indication for rehabilitation may have affected our results, this finding nevertheless indicates that even severe COVID-19 cases may be treated with inpatient rehabilitation for functional recovery.

To the best of our knowledge, this is the first study of a rehabilitation protocol for COVID-19 at a Japanese institution. Although severe pulmonary injury may affect the length of hospital stay and ADL, comprehensive rehabilitation, including musculoskeletal and respiratory rehabilitation, may be useful for patients with COVID-19 to prevent disuse during this period. A previous study^8^ proposed a rehabilitation protocol that comprised only two intervention programs for two categories of patients. In the current study, the enrolled patients underwent a more stringent rehabilitation protocol than that used in the aforementioned study.^8^ In addition, our rehabilitation protocol defines the criteria for exercise therapy in more detail than previously proposed protocols. Our rehabilitation protocol suggests the possibility of providing a step-by-step rehabilitation program for both severe and mild cases. We believe that our protocol provides a benchmark for the COVID-19 rehabilitation program and ADLs at discharge from an acute setting.

Our findings regarding the influence of COVID-19 on ADLs are consistent with those of previous studies.^9,10^ Notably, many patients are discharged when their ADLs have not yet fully recovered, and the need for post-acute care is high, as mentioned in a previous study.^16^ It has also been found that functional status is a strong predictor of the discharge destination for patients with COVID-19.^27^ There are two possible reasons for the decline in ADL: 1) pulmonary dysfunction and 2) disuse associated with hospitalisation and treatment. Efforts to prevent disuse during hospitalisation can be made through rehabilitation. Thus, it is important to identify patients with COVID-19 who are likely to experience a decline in ADLs from the early stage of hospitalisation and to provide them with intensive rehabilitation. Continuation of rehabilitation after discharge from acute care settings is also required.

The current study involved several limitations. First, this was a single-centre retrospective study conducted in Japan. Therefore, the generalisability of these results to other countries and institutions should be considered with caution, particularly the criteria for discharge from hospital, which can affect outcomes and may vary from one hospital to another. Additionally, this investigation employed the Japanese COVID-19 severity classification, a methodology that diverges from studies anchored on other international criteria. To ensure a more comprehensive understanding of COVID-19 rehabilitation in acute care, reports from multiple hospitals are needed. Second, the validity of the protocol and level transition criteria have not yet been established. Third, in this study, we focused on activity in terms of the International Classification of Functioning domain; however, it will be necessary to consider other domains, such as function and participation, in the future. Fourth, we were unable to examine ADL prior to COVID-19. As such, it is possible that patients with low ADL prior to the outset of COVID-19 may have been included, thereby affecting the results. Furthermore, the absence of a comparative analysis between status at admission and discharge, coupled with the descriptive nature of the study and the lack of a control group, constitute significant methodological limitations. Thus, the current study did not show the effect of rehabilitation directly. Fifth, we were not able to examine the influence of ICU-acquired weakness. In general, longer durations of mechanical ventilation and hospitalisation result in higher functional impairment for survivors.^28^ It is also possible that ICU-acquired weakness contributed to poor patient outcomes at discharge. Sixth, we did not identify any factors associated with poor functional outcomes. However, the sample size in this study was insufficient for multiple analyses. Future studies will be needed to identify the factors associated with functional outcomes. Finally, all patients in this study contracted COVID-19 in the early stages of the pandemic in Japan, when vaccine uptake was still low, particularly among young people. In addition, because the ICU and acute care units were in a university hospital, we may have received patients who were in a relatively serious condition. In the severe group, there is a possibility that many young patients with risk factors, such as obesity,^29^ were included. As such, the symptoms and severity of COVID-19 in the future may differ from those outlined in this study.

In conclusion, we found that severity was a significant factor in the functional outcome at discharge among patients hospitalised for COVID-19. Although the prognosis of patients with COVID-19 has changed because of mutations in the virus and the widespread use of vaccines, our rehabilitation protocol and associated findings may apply to other infectious disease outbreaks. Further studies will be required to examine the validity and effectiveness of our rehabilitation protocol for patients with various SARS-CoV-2 variants and other viruses.

## Conflicts of Interest

The authors have no conflicts of interest to declare. This research received no specific grants from any funding agency in the public, commercial, or not-for-profit sectors.

## Figures and Tables

**Table1:** Overview of the rehabilitation protocol for COVID-19

	Level 1 (Sedation)	Level 2 (Sitting Up)		Level 3 (Standing)	Level 4 (Walking)	Level 5 (Aerobic exercise)	Level 6 (High-intensity exercise)	Level 7 (Gym exercise)
Place	Intensive care unit			Bed		Rehabilitation centre		

Treatment	ECMO^a^, Ventilator, blood purification, prone position	ECMO^a^, Ventilator, blood purification	Ventilator, Tracheostomy, oxygen therapy	Tracheostomy, oxygen therapy				

Rehabilitation	(Collaboration with nurse) Position change, functional position maintenance, NMES^b^	(Collaboration with nurse) Position change, range of motion exercises, early mobilisation and NMES^b^	Range of motion exercises, respiratory exercises, stretching, early mobilisation, NMES^b^	Respiratory exercises in the supine position, muscle strengthening, stretching (respiratory muscles, extremities), sitting upright, balance exercises, slow walking	Respiratory muscle stretching, muscle strengthening, walking *Gradually increase activity by 10–15 min/day	Two sets of 5-minute aerobic exercise (walking at a comfortable speed, ergometer, stair climbing, etc.) *Gradually increase by one set if possible.	High-intensity aerobic exercise (fast walking, running, side stepping, etc.)	Gym training (running, strength training under dead weight load, etc.)

Standards of rehabilitation implementation	Stabilisation of vital signs RPE^c^: 6–8	RPE^c^: 6–8 RR^d^ <30/min SpO2 drop of 5% or less and maintaining 88%	RPE^c^: 6–10 RR^d^ <30/min, SpO2 drop of 5% or less and maintaining 88%	RPE^c^: 6–11 RR^d^ <30/min SpO2 drop of 5% or less and maintaining 88%	RPE^c^: 12–14 RR^d^ <30/min SpO2 drop of 5% or less and maintaining 88%	RPE^c^: 12–14	RPE^c^ >15	

Criteria for stepping up in level	Management of awake patients	Weaning from ECMO^a^, or RPE^c^ 6–8 in sitting position	Patient is able to sit in RPE^c^ 6–10	Patient can walk 200 m with RPE^c^ less than 11, or if the patient meets the above criteria for 3 consecutive days	Minimum of 7 days 40-minute sessions of aerobic rehabilitation, or when the patient is able to recover from symptoms of fatigue within 1 hour	Normal fatigue level		

^a^ Extracorporeal membrane oxygenation; ^b^ neuromuscular electrical stimulation; ^c^ rate of perceived exertion; ^d^ respiratory rate.

**Table2:** Baseline characteristics of the study participants

	Mild (n=1)	Moderate I (n=6)	Moderate II (n=41)	Severe (n=30)	*p*-value^a^	Post hoc test^b^		
						Moderate I vs. Moderate II	Moderate I vs. Severe	Moderate II vs. Severe
Age, mean (SD^a^)	83.0 (0.0)	85.7 (5.8)	72.2 (13.5)	64.3 (12.9)	0.001	0.014	<0.001	0.014
Sex; male, number	0	2	26	21	0.213	—	—	—
Body mass index on admission, kg/m^2^, mean (SD^a^)	23.9	18.8 (2.7)	23.0 (6.3)	28.7 (6.7)	<0.001	0.024	0.002	0.001
Number of patients admitted to the intensive care unit, n	0	0	33	30	—			

Comorbidity, n (%)					—			
Malignant tumour	0 (0)	0 (0)	10 (24.4)	4 (13.3)				
Chronic obstructive pulmonary disease	0 (0)	3 (50.0)	4 (9.8)	1 (2.5)				
Other lung disease	0 (0)	1 (16.7)	1 (2.4)	4 (13.3)				
Chronic kidney disease	1 (100.0)	1 (16.7)	5 (12.2)	3 (10.0)				
Diabetes mellitus	0 (0)	0 (0)	15 (36.6)	9 (30.0)				
Hypertension	0 (0)	2 (33.3)	23 (56.1)	14 (46.7)				
Dyslipidaemia	0 (0)	1 (16.7)	11 (26.8)	9 (30.0)				
Obesity (body mass index >30)	0 (0)	0 (0)	3 (7.3)	9 (30.0)				
Smoking	0 (0)	0 (0)	3 (7.3)	5 (16.7)				
Cardiovascular disease	0 (0)	2 (33.3)	8 (19.5)	3 (10.0)				
Stroke	0 (0)	1 (16.7)	1 (2.4)	3 (10.0)				

SD, standard deviation. ^a^ The comparison was performed excluding the single case of mild severity. ^b^ When statistically significant between-group differences were found (*p*<0.05), multiple comparisons among all groups were performed using the Bonferroni correction.

**Table3:** Comparison of the functional independence measure score at discharge according to COVID-19 severity classification in all participants

	Mild (n=1)	Moderate I (n=6)	Moderate II (n=41)	Severe (n=30)	*p*-value^c^	Post hoc test^d^		
						Moderate I vs. Moderate II	Moderate I vs. Severe	Moderate II vs. Severe
FIM score at discharge, mean (SD^a^)								
Motor subtotal	85.0	47.8 (25.2)	54.2 (30.1)	31.6 (30.4)	0.027	0.056	0.288	0.030
Cognitive subtotal	34.0	20.0 (7.3)	25.3 (13.2)	16.7 (12.9)	0.026	0.258	0.355	0.034
Length of hospital stay, days, mean (SD^a^)	22.0	23.0 (8.3)	25.8 (14.0)	45.7 (17.7)	<0.001	0.898	0.004	<0.001
Days of rehabilitation period, days, mean (SD^a^)	20.0	9.5 (4.3)	17.0 (10.2)	29.6 (19.7)	<0.001	0.089	0.054	<0.001
Total rehabilitation time, minutes, mean (SD^a^)	720.0	286.7 (150.8)	495 (425.3)	1468 (1232.1)	<0.001	0.232	0.016	<0.001
Final level of the rehabilitation protocol, median (IQR^b^)	3	3 (3–3.75)	3 (3–3)	2 (2–3)	0.002	0.848	0.075	0.003
Discharge destination, n								
Home	1	2	19	3	0.012	0.497	0.336	0.029
Other	0	4	12	13				
Death	0	0	10	14				

^a^ standard deviation; ^b^ interquartile range. ^c^ The comparison was performed excluding the single case of mild severity. ^d^ When statistically significant between-group differences were found (*p*<0.05), multiple comparisons among all groups were performed using the Bonferroni correction.

**Table4:** Comparison of the functional independence measure score at discharge and rehabilitation period according to COVID-19 severity classification in survivors

	Mild (n=1)	Moderate I (n=6)	Moderate II (n=31)	Severe (n=16)	*p*-value^c^	Post hoc test^d^		
						Moderate I vs. Moderate II	Moderate I vs. Severe	Moderate II vs. Severe
FIM score at discharge, mean (SD^a^)								
Motor subtotal	85.0	47.8 (25.2)	67.5 (21.8)	47.8 (34.2)	0.154	—	—	—
Cognitive subtotal	34.0	20.0 (7.3)	31.9 (7.4)	26.9 (9.3)	0.002	0.001	0.130	0.098
Length of hospital stay, days, mean (SD^a^)	22.0	23.0 (8.3)	25.8 (14.1)	40.8 (17.5)	0.004	0.999	0.027	0.007
Days of rehabilitation period, days, mean (SD^a^)	20.0	9.5 (4.3)	10.8 (7.7)	23.7 (18.8)	0.004	0.082	0.023	0.024
Total rehabilitation time, minutes, mean (SD^a^)	720.0	286.7 (150.8)	535.5 (459.4)	1632.5 (1342.7)	0.002	0.187	0.016	0.006
Final level of the rehabilitation protocol, median (IQR^b^)	3	3 (3–3.75)	3 (3–4)	3 (2.75–4)	0.263	—	—	—
Discharge destination, n								
Home	1	2	19	3	0.047	0.999	0.999	0.084
Other	0	4	12	13				

^a^ standard deviation; ^b^ interquartile range. ^c^ The comparison was performed excluding the single case of mild severity. ^d^ When statistically significant between-group differences were found (*p*<0.05), multiple comparisons among all groups were performed using the Bonferroni correction.

**Table5:** Functional independence measure scores at discharge according to the rehabilitation level

	Level 1 (n=1)	Level 2 (n=3)	Level 3 (n=28)	Level 4 (n=18)	Level 5 (n=3)	Level 6 (n=1)	*p*-value^c^	Post hoc test^d^					
								Level 2 vs. Level 3	Level 2 vs. Level 4	Level 2 vs. Level 5	Level 3 vs. Level 4	Level 3 vs. Level 5	Level 4 vs. Level 5
FIM score at discharge, mean (SD^a^)													
Motor subtotal score	13	29.7 (12.7)	51.3 (34.9)	75.6 (15.1)	81.1 (35.8)	91	0.004	0.483	0.067	0.243	0.037	0.209	0.483
Cognitive subtotal score	12	26.7 (8.5)	26.2 (9.9)	33.7 (3.5)	35 (0)	35	0.014	0.999	0.299	0.590	0.031	0.380	0.999
Length of hospital stay, days, mean (SD^a^)	39	31.0 (8.5)	30.8 (18.2)	24.3 (11.6)	48.0 (14.9)	38	0.119	—	—	—	—	—	—
Days of rehabilitation period, days, mean (SD^a^)	36	17.0 (3.3)	15.1 (16.6)	9.2 (5.9)	23.3 (9.4)	23	0.233	—	—	—	—	—	—
Total rehabilitation time, minutes, mean (SD^a^)	1340	1086.7 (288.6)	876.4 (1177.5)	518.9 (527.6)	1546.7 (563.2)	2040	0.041	0.306	0.196	0.765	0.765	0.306	0.109
Discharge destination, n													
Home	—	—	14	11	2	—	0.238	—	—	—	—	—	—
Others	1	3	14	7	1	1							

^a^ SD, standard deviation. ^c^ The comparison was performed for levels 2–5, excluding levels 1 and 6. ^d^ When statistically significant between-group differences were found (*p*<0.05), multiple comparisons among all groups were performed using the Bonferroni correction.
