# Supplementary material for: Functional outcomes of protocol-based rehabilitation for patients with coronavirus disease 2019 in an acute care setting
Source: Fujita Med J. 2023 Nov 29;10(1):35–42. doi: 10.20407/fmj.2023-015 (PMC10847633; doi:10.20407/fmj.2023-015)
Supplement: Supplementary file 1 — Supplementary Tables [file fmj-10-035-s001.pdf]

Supplementary table 1. Severity definition of Novel Coronavirus Infection COVID-19 Medical Practice Guidelines Version 5.3

Severity	Oxygen saturation	Clinical manifestation
Mild	$SpO_2 \geq 96\%$	No respiratory symptoms or only cough without dyspnea<br>No findings of pneumonia
Moderate I	$93\% < SpO_2 < 96\%$	Dyspnea, no findings of pneumonia
Moderate II	$SpO_2 \leq 93\%$	Requires oxygen therapy
Severe		Admission to the intensive care unit or requires ventilator

SpO_2, saturation of percutaneous oxygen

Supplementary table 2. Survivors baseline characteristics

	Mild (n = 1)	Moderate I (n = 6)	Moderate II (n = 31)	Severe (n = 16)	p-value*	Post-hoc test** Moderate I vs. Moderate II	Moderate I vs. Severe	Moderate II vs. Severe
Age, mean (SD)	83.0 (0.0)	85.7 (5.8)	72.2 (13.5)	64.3 (12.9)	0.001	0.009	0.002	0.108
Gender; male, number	0	2	21	11	0.247	-	-	-
Body mass index on admission, kg/m^2, mean (SD)	23.9	18.8 (2.7)	24.4 (8.3)	30.0 (7.5)	<0.001	0.001	0.004	0.015
Number of patients who were admitted to the intensive care unit, n	0	0	24	16	-			
Comorbidity, n (%)					-			

Malignant tumor	0 (0)	0 (0)	4 (12.9)	2 (12.5)
Chronic obstructive pulmonary disease	0 (0)	3 (50.0)	3 (9.7)	0 (0)
Other lung disease	0 (0)	1 (16.7)	1 (3.2)	2 (12.5)
Chronic kidney disease	1 (100.0)	1 (16.7)	4 (12.9)	2 (12.5)
Diabetes mellitus	0 (0)	0 (0)	12 (38.7)	6 (37.5)
Hypertension	0 (0)	2 (33.3)	17 (54.8)	10 (62.5)
Dyslipidaemia	0 (0)	1 (16.7)	7 (22.6)	4 (25.0)
Obesity（body mass index > 30)	0 (0)	0 (0)	3 (9.7)	5 (31.3)
Smoking	0 (0)	0 (0)	3 (9.7)	2 (12.5)
Cardiovascular disease	0 (0)	2 (33.3)	7 (22.6)	2 (12.5)
Stroke	0 (0)	1 (16.7)	1 (3.2)	2 (12.5)

SD, standard deviation.

*The comparison was performed excluding single case of mild severity.

**When statistically significant between-group differences were found ($p < 0.05$), multiple comparisons between all groups were performed using the Bonferroni correction.
